# Exploring the Relationship between COVID-19 Vaccine Refusal and Belief in Fake News and Conspiracy Theories: A Nationwide Cross-Sectional Study in Italy

**DOI:** 10.3390/ijerph19159350

**Published:** 2022-07-30

**Authors:** Giuseppina Lo Moro, Giacomo Scaioli, Fabrizio Bert, Andrea Lorenzo Zacchero, Ettore Minutiello, Roberta Siliquini

**Affiliations:** 1 Department of Public Health Sciences and Pediatrics, University of Turin, 10126 Turin, Italy; giuseppina.lomoro@unito.it (G.L.M.); giacomo.scaioli@unito.it (G.S.); andrea.zacchero@edu.unito.it (A.L.Z.); ettore.minutiello@unito.it (E.M.); roberta.siliquini@unito.it (R.S.); 2 AOU City of Health and Science of Turin, 10126 Turin, Italy

**Keywords:** vaccine refusal, pandemic, infodemic, fake news, conspiracy theories

## Abstract

The COVID-19 pandemic has been accompanied by an infodemic, which includes fake news (FNs) and conspiracy theories (CTs), and which may worsen vaccine refusal (VR), thus hindering the control of the transmission. This study primarily aimed to assess COVID-19 VR in Italy and its relationship with belief in FNs/CTs. Secondarily, it explored the conviction in FNs and CTs and associated variables. An online cross-sectional study was conducted in Italy (2021). The primary outcome was VR and secondary outcomes were FN misclassification score (0% to 100%: higher score means higher misclassification) and CT belief score (1 to 5: higher score means higher agreement). There were 1517 participants; 12.3% showed VR. The median FN and CT scores were: 46.7% (IQR = 40–56.7%) and 2.8 (IQR = 2.2–3.4). Age, education, FN, and CT scores had significant associations with VR. Education, economic situation, health and e-health literacy showed significant relationships with secondary outcomes. Study/work background had a significant association only with the FN score. FN and CT scores were associated. This work estimated a VR lower than before the first COVID-19 vaccine approval. The relationship between VR and FN/CT belief represents a new scenario, suggesting the need for planning effective strategies to tackle FNs and CTs to implement successful vaccination campaigns.

## 1. Introduction

Vaccine hesitancy is a complex phenomenon that includes indecision, delay, and reluctance and refers to delayed acceptance or refusal of vaccination despite the availability of vaccination facilities [1]. Indeed, although vaccinations are considered as one of the most relevant successes in public health, a growing number of individuals have been reluctant toward this measure for a long time [2], and the World Health Organization (WHO) recognized vaccine hesitancy to be in the top ten threats to global health [3]. It is worth noting that vaccine hesitancy can vary considering the type of vaccine [2]. Specifically, individuals may be more skeptical towards vaccines that are more recently introduced and used for less time [2]. Against this background, it seems clear that in the context of SARS-CoV-2 pandemic it is essential to study vaccine hesitancy evaluating prevalence and determinants. Indeed, the fast approval of the first vaccines [4,5] represented an extraordinary situation that may have a role in population’s vaccine acceptance.

In the European context, Italy may represent a particularly hesitant country. Indeed, before the pandemic, a study involving 67 countries showed that Italy was among the countries with the highest level of skepticism and doubt regarding the effectiveness of vaccinations [6]. Then, during the first year of the pandemic, two relevant systematic reviews highlighted that Italy was among the countries with the lowest COVID-19 vaccine acceptance worldwide (Italy was among the last seven countries for acceptance, with 53.7% of acceptance) [7] and in Europe (Italy was at the last position, with 40.9% of acceptance) [8]. Moreover, an international study involving 19 countries revealed that Italy was at the 11th position for COVID-19 vaccine acceptance [9]. Lastly, it has been studied that COVID-19 vaccine acceptance had a declining trend in Italy during 2020, both considering repeated cross-sectional and longitudinal studies [10,11].

In these extraordinary circumstances, it has been highlighted that a possible relationship exists between negative information on COVID-19 vaccines seen on social media and low acceptance, suggesting a relevant role played by disinformation [11]. Indeed, the pandemic has been accompanied by an unprecedented infodemic, i.e., an overabundance of information that makes it hard for people to find trustworthy sources and reliable guidance when they need it. Such information can be inaccurate: the infodemic can contribute to the spread of fake or altered news and of conspiracy theories [12,13]. Specifically, the infodemic can worsen the impact of the pandemic by hindering the search for reliable sources, making people feel overwhelmed and influencing the decision-making process [12,13]. Although this field of research is continuously evolving [13], some studies started to deepen this topic, suggesting that infodemic, disinformation and conspiracy theories may impact individuals’ health, not only fueling fears [14] among a mentally worn out population [15,16], but also making people believe that preventive measures are not useful [17] and leading to low adherence to measures implemented to limit COVID-19 transmission [18,19,20,21]. Concurrently, some authors have proposed theoretical frameworks to explain the impact of fake news and conspiracy theories on vaccine hesitancy [22,23]. Therefore, due to the substantial role of vaccines in limiting the spread of the infection, it seemed compelling to deepen the study of the potential influence of fake news and conspiracy theories on vaccine intention. In particular, in the Italian context there were no works examining the ability to distinguish fake news or the belief in conspiracy theories in relation with vaccine acceptance or refusal after the approval of the first COVID-19 vaccine, with the exception of a study surveying a sample of university students that found an association between hesitancy and agreement with conspiracy statements [24].

Thus, the present study primarily aimed to assess the prevalence of COVID-19 vaccine refusal in Italy and its relationship with belief in fake news and conspiracy theories. Secondarily, another aim was to explore the extent of the conviction in fake news and conspiracy theories and the variables that were potentially associated with these infodemic-related phenomena.

## 2. Materials and Methods

### 2.1. Study Design, Sampling, and Questionnaire

Between 12 April 2021 and 16 May 2021, a cross-sectional survey was performed in Italy through the Computer-Assisted Web Interview (CAWI) method. The Bioethics Committee of the University of Turin approved the study. Raosoft^®^ was used to determine that the minimum sample size was 385, based on a 5% margin of error, 95% confidence level, 50% response distribution and size of the population living in Italy. An anonymous online questionnaire was set on the LimeSurvey platform and was distributed via the main social networks (e.g., Instagram and Facebook). The inclusion criteria for the recruitment were an age of 18 years or more, an understanding of Italian language, and residence in Italy. Participants gave their informed consent and received no compensation.

First, the questionnaire included items on sociodemographic information, such as age, gender, nationality, education level, perceived economic situation, and main occupation. The job/study background was further investigated, with attention to the field of health care. In addition, participants were asked whether they had close friends or family members who are health care workers (HCWs), whether they had a chronic disease, and whether they lived with/took frequently care of a frail person (e.g., older or vulnerable people). Also, the survey included pandemic-related items by asking participants if they had contracted COVID-19 and if they had relatives or close friends who had contracted it and by exploring vaccine refusal.

Specifically, vaccine refusal (i.e., the primary outcome of the present paper) was investigated by asking participants if they had received at least one dose of COVID-19 vaccine and if, in case of a negative response, they intended to undergo vaccination when they had the chance. Thus, participants reporting vaccine refusal were identified if they did not get the vaccine and intended to refuse it, as measured in other studies [25].

Then, HL and eHL were assessed by using the Italian version of two validated scales: the Single Item Literacy Screener (SILS) [26,27] and the eHealth Literacy Scale (eHEALS) [28,29]. The SILS was used to screen self-reported HL levels. It consists of one item that identifies the need for help when reading instructions or other written material provided from doctors or pharmacy. A score greater than 2 represents inadequate HL [26,27]. The eHEALS is an eight-item tool that assesses participants’ self-reported knowledge and competence in researching, evaluating, and applying health information found online [28,29]. It is possible to define inadequate eHL when the total eHEALS score is less than 26 [30,31].

Last, the secondary outcomes were explored, i.e., misclassification of fake news and belief in conspiracy theories. The misclassification of fake news was investigated by asking participants to identify if 20 statements were true or false. Among these statements, fake news were mainly selected from the website of the Italian Ministry of Health [32]. The authors assigned one point for each statement that was misclassified and, then, the percentage of wrong classification was calculated. Thus, the score of misclassification ranged from 0% to 100%; a higher score represents a worse identification of fake news. Details on the items of this score can be find in Supplementary Material S1 (Table S1). The belief in conspiracy theories was assessed by presenting five statements from a WHO tool, developed to conduct behavioral insights studies related to COVID-19 [33]. Participants were asked to indicate the level of agreement (from 1, “totally disagree”, to 5, “totally agree”) with these five conspiracy theories. The total score was calculated by averaging the responses to the five items [30]; therefore it ranged from 1 to 5. As the score increases, the conviction in such theories is higher. Details on the items of this score can be found in Supplementary Material S1 (Table S2).

### 2.2. Statistical Analysis

Descriptive analyses were carried out for all variables. Age and scores of misclassification of fake news and belief in conspiracy theories were expressed as median and interquartile range (IQR) because the Shapiro–Wilk test indicated that these data had non-normal distributions.

Then, differences in the distribution of the outcomes across the variables of the questionnaire were explored through chi-squared tests and the Mann–Whitney U test (or Kruskal–Wallis test when appropriate).

Lastly, to explore variables associated with the outcomes, univariable and multivariable models were carried out (logistic regressions for vaccine refusal and linear regressions for secondary outcomes). The multivariable models, adjusted for age, gender, and educational level, were achieved with a stepwise forward selection process, with a univariable *p*-value < 0.250 as the main criterion [34]. Results were expressed as odds ratio (OR) (logistic regression), unstandardized coefficients (Coef.) (linear regression), and 95% confidence interval (95% CI).

Stata (v16) was used, and a two-tailed *p*-value < 0.05 was considered statistically significant. Missing values were excluded.

## 3. Results

### 3.1. Descriptive Analyses

The final sample consisted of 1517 participants (77.7% females; median age 41 years, IQR = 28–54). The characteristics of the sample are described in Table 1 and Table 2. In particular, 57.8% declared to have a high school diploma or a lower educational level and 24.2% stated to work/study in a health care field. A total of 16.5% proved to have an inadequate HL and 41.6% an inadequate eHL. Lastly, 15.4% had personally contracted SARS-CoV-2, and 74.1% had relatives or close friends who had contracted it.

Regarding the primary outcome, 186 individuals (12.3%) reported COVID-19 vaccine refusal. Considering the secondary outcomes, the median of the score of fake news misclassification was 46.7% (IQR = 40–56.7%), and the median of the score of belief in conspiracy theories was 2.8 (IQR = 2.2–3.4).

The fake news that was misclassified more frequently were as follows: “taking vitamins C and D prevents SARS-CoV-2 infection” (69.5% of misclassification), “the effectiveness of the mask is not affected by the length of the beard” (68.7%), and “ibuprofen administration in patients who have developed COVID-19 significantly worsens the disease” (55.0%). All details are shown in Supplementary Material S1 (Table S1). The conspiracy theories with the highest degree of agreement were as follows: “politicians usually do not tell us the true motives for their decisions” (median 4, IQR = 3–5) and “many very important things happen in the world, which the public is never informed about” (median 3, IQR = 3–5). Details in Supplementary Material S1 (Table S2).

Considering the chi-squared analyses, certain variables resulted in association with COVID-19 vaccine refusal (Table 1 and Table 2). For instance, a greater prevalence of refusal was reported by participants with high school diploma or lower educational level (16.7%), individuals with insufficient/poor economic situation (22.1%), people who did not have a relative/close friend working in health care (13.7%), and participants who did not share their habitation with a frail individual (13.4%) (Table 1). Moreover, the prevalence of vaccine refusal was significantly higher among participants who had relatives/close friends who had contracted SARS-CoV-2 (16.4%). All secondary outcomes were associated with vaccine refusal (Table 2). Lastly, the distribution of the secondary outcomes was significantly different across the categories of educational level, background of work/study, economic situation, and HL (Supplemental Material S1, Table S3).

### 3.2. Regression Analyses

Considering the multivariable model, several variables resulted to be significantly associated with the primary outcome vaccine refusal (Table 3). Indeed, participants with higher age, students, and those who shared habitation with a frail individual (or cared for a frail individual daily) reported a lower likelihood of vaccine refusal. People with a high school diploma or a lower educational level had a higher likelihood instead. The higher were the scores of the secondary outcomes, the higher was the probability of showing vaccine refusal.

Regarding the secondary outcomes, multivariable models are shown in Table 4 and Table 5. Having a high school diploma or a lower educational level, an insufficient/poor economic situation, and an inadequate HL according to SILS were positively associated with both the scores. Having a study/work background different from health care and not being a worker had a positive association only with the fake news misclassification score, whereas being female had a positive association only with the belief in conspiracy score. Having a relative/close friend who had contracted SARS-CoV-2 had a negative association only with the fake news misclassification score. The eHL (according to the eHEALS) showed a different relationship with the two scores. Indeed, an inadequate eHL was positively associated with the fake news misclassification score and negatively associated with the belief in conspiracy theories score. Lastly, the two secondary outcomes were positively associated with each other.

## 4. Discussion

The present paper had the primary aim of studying the prevalence of COVID-19 vaccine refusal in Italy and its relationship with belief in fake news and conspiracy theories. Secondarily, it aimed to describe convictions in fake news and conspiracy theories and the factors that were potentially associated with such beliefs.

We found a COVID-19 vaccine refusal of 12%. Compared with data collected before the approval of the first vaccine by the Food and Drug Administration (FDA) and the European Medicines Agency (EMA) [4,5], this study showed a lower prevalence. Indeed, a meta-analysis calculated a percentage of refusal of 20% (95% CI: 13–29%) [25]. Furthermore, the results of other studies conducted in Italy in the first months of 2020 revealed a refusal or hesitancy of 29.5% [35] and 41% [36]. Similarly, considering that 88% of our sample underwent or would undergo vaccination, our data showed an increase in acceptance compared with pre-approval reviews that reported Italy among the countries of the world and Europe with lower COVID-19 vaccine acceptance (53.7% [7] and 40.9% [8]). Therefore, although some authors have reported a declining trend of acceptance of the COVID-19 vaccine in Italy during 2020 [10,11], it is possible that, after the approval of the first vaccine, hesitancy has decreased. This finding of a potential reduction in VR after the first COVID-19 vaccine approval seems to be confirmed by a study on vaccine hesitancy among Italian medical students [37]. Indeed, although the VR found among medical students was substantially lower than the VR of our study, the observation period included the date of approval of the first vaccine by the EMA, and it was reported that participants who completed the questionnaire after that date had a significantly lower probability of being hesitant. Lastly, post-approval studies conducted in Italy involving the general population showed a wide range of results, reporting a COVID-19 vaccine acceptance of 75.6% [38] and a prevalence of hesitancy from 31% [39] to 12% [40].

Regarding the main socio-demographic characteristics associated with a higher vaccination hesitancy, the results of this study confirmed findings from relevant systematic reviews. Specifically, most studies suggested an association between COVID-19 vaccine hesitancy and a low level of education or a young age [11,25,41,42]. Other factors such as employment and willingness to protect loved ones who belong to frail groups have also been identified in other studies for their role in hesitancy [41]. It worth noting that neither the study/work background (with attention to belonging to the HCWs category) nor the HL or eHL were associated with vaccine refusal. Although it could be assumed that HCWs and participants with higher HL were less likely to report hesitancy, both of these characteristics have actually revealed conflicting associations with vaccine hesitancy in scientific literature, and further investigation is needed [11,43]. Last but not least, it is important to underline the significant association between vaccine refusal and the secondary outcomes (i.e., the score of fake news misclassification and belief in conspiracy theories), which was the main focus of this work. These relationships, probably amplified by the infodemic, represented a novelty in the vaccine hesitancy scenario and have still been examined in a few studies [24,44,45], thus suggesting an urgent need to study and plan effective strategies to tackle fake news and conspiracy theories to achieve successful vaccination campaigns.

The study on the two secondary outcomes was therefore of particular interest. First of all, it is worth noting that the median score for fake news misclassification was 46.7%, suggesting a poor ability in classifying news and a low level of information on SARS-CoV-2. Secondly, some of the most frequently misclassified fake news could lead to health damage. For instance, nearly 70% of our sample stated that vitamins C and D should be taken to prevent COVID-19. However, an improper use of dietary supplements can be potentially dangerous, as highlighted in a document published in 2020 by the Italian National Institute of Health following the increased interest in supplements during the pandemic [46]. Other fake news could also have negative implications on behaviors, such as the belief that wearing a mask can lead to carbon dioxide poisoning or that holding your breath for 10 s can rule out SARS-CoV-2 infection, respectively. These beliefs were not recognized as fake news by 29.4% and 25.4% of participants. Lastly, the most frequently misclassified fake news corresponded in part to the fake news most shared on social media according to some authors [47]. Regarding conspiracy theories, these can spread when people feel more anxious and unable to control their future [48] and can lead to a high degree of distrust in authorities, as underlined by the conspiracy theory with a higher degree of agreement in our study. Specifically, it was reported that distrust of authorities, especially politicians, increased during the pandemic [49], potentially causing not only political but also economic and health damage [50,51,52].

Furthermore, some variables were associated with the increase of both the secondary outcomes, e.g., lower education level and poor/insufficient economic situation, i.e., factors already identified by other studies as associated with poor recognition of incorrect information [53,54,55]. It is interesting to note that our study showed a significant association for work/study background only with the score of fake news misclassification and not with the score of belief in conspiracy theories, thus suggesting that being an HCW may be protective only in the recognition of the statements related to the spread and prevention of COVID-19 and not in the recognition of statements related to the socio-political situation.

It is also worth noting that gender had a significant association only with belief in conspiracy theories. Because it has been found that anxiety levels correlate with higher propensity to believe in conspiracy theories [55] and women may have reported higher levels of anxiety and concerns due to the pandemic [56,57], further studies should consider evaluating the relationship between gender, anxiety, and belief in conspiracy theories.

In addition, inadequate HL was associated with an increase in the two secondary outcome scores, as similarly suggested by other authors [47]. Conversely, eHL reported conflicting relationships with the two outcomes, showing a positive association with fake news misclassification and a negative association with belief in conspiracy theories. These findings may suggest that it is probable that people who were unable to use the information found on the internet for their own health could not actually discriminate between true and false news. However, as an opposite relationship has turned out with regard to conspiracy theories, it is possible that, because the web is the main means of sharing and disseminating conspiracy theories, individuals who were unable to exploit these technological resources were even less exposed to certain theories and therefore less inclined to believe what was proposed in the questionnaire.

Lastly, it should be noted that the two secondary outcomes were significantly associated with each other, showing a strong relationship and potentially common determinants that deserve to be further investigated in future studies.

The present study had some limitations that should be acknowledged. Among the main limitations of the study, it is necessary to highlight the cross-sectional design, which only allows us to formulate hypotheses but not to establish causal links, and opportunistic sampling. In addition, the choice of using a survey instead of in-depth interviews may limit the research results. Furthermore, the distribution of the questionnaire via social media may have excluded a certain subpopulation, and no information was collected on individuals who refused to participate in the study. In addition, the prevalence of foreign people participating in the study was lower than the one of foreign people living in Italy, partially because the questionnaire was only in Italian [58]. Similarly, the prevalence of women was higher than the one in Italy [58], consistently with findings showing that male participants may have lower response rates [59]. Therefore, the results cannot be generalized to the entire population living in Italy. Finally, vaccine refusal may be different for distinct COVID-19 vaccines, as reported for other types of vaccines [2]. However, the study represented a novelty in the Italian context and can provide useful data with which to explore this unprecedented situation and plan future studies on strategies to tackle fake news and conspiracy theories to achieve successful vaccination campaigns.

## 5. Conclusions

The present paper estimated an COVID-19 vaccine refusal lower than the prevalence detected before the approval of the first vaccine by the FDA and EMA. Among the several variables that were significantly associated with a greater probability of vaccine refusal, it is worth highlighting the association with a poor ability to recognize fake news and the belief in some conspiracy theories. These relationships, probably amplified by the infodemic, represent a novelty in the vaccine hesitancy scenario, thus suggesting an urgent need to plan effective strategies to address fake news and conspiracy theories to achieve successful vaccination campaigns. Therefore, together with the pandemic, a further challenge arises. The research agenda to address this challenge, as defined by the WHO, offers many ideas for researchers worldwide, who can contribute to the solution by measuring the impact of the infodemic, detecting the spread of the infodemic, testing and evaluating interventions that protect against infodemics and mitigate negative effects, and promoting the development, adaptation, and application of shared tools [13].

## Figures and Tables

**Table 1:** Sociodemographic characteristics of the sample and relationship with vaccine refusal.

Characteristic	Overall Sample	COVID-19 Vaccine Refusal		
	(n = 1517) N (%)	No (n = 1327) N (%)	Yes (n = 186) N (%)	*p*-Value
Age *	41 (28–54)	42 (28–54)	40 (33–52)	0.690
**Gender**				
Male	338 (22.3)	304 (89.9)	34 (10.1)	0.152
Female	1176 (77.7)	1020 (87)	152 (13)	
**Nationality**				
Italian	1469 (96.9)	1289 (88.0)	176 (12.0)	0.057
Other	47 (3.1)	37 (78.7)	10 (21.3)	
**Educational level**				
Higher education	639 (42.2)	597 (93.7)	40 (6.3)	**<0.001**
High school diploma or lower	877 (57.8)	730 (83.3)	146 (16.7)	
**Background (study/work)**				
Healthcare	367 (24.2)	345 (94.5)	20 (5.5)	**<0.001**
Informatics	149 (9.8)	126 (84.6)	23 (15.4)	
Journalism	19 (1.3)	17 (89.5)	2 (10.5)	
Other	979 (64.7)	837 (85.6)	141 (14.4)	
**Occupation**				
Worker	1023 (67.6)	888 (86.9)	134 (13.1)	**0.002**
Student	194 (12.8)	184 (95.3)	9 (4.7)	
Other	296 (19.6)	254 (85.8)	42 (14.2)	
**Economic situation**				
Excellent/good	1182 (78.1)	1069 (90.4)	113 (9.6)	**<0.001**
Insufficient/poor	332 (21.9)	258 (77.9)	73 (22.1)	
**Relative/close friend working in health care**				
No	962 (63.5)	830 (86.3)	132 (13.7)	**0.025**
Yes	552 (36.5)	497 (90.2)	54 (9.8)	
**Sharing the habitation with a frail individual °**				
No	1179 (78)	1021 (86.6)	158 (13.4)	**0.010**
Yes	333 (22)	305 (91.9)	27 (8.1)	

*p*-value obtained via chi-squared test (significant *p*-values in bold). Figures expressed as frequency and percentage (column percentage in the overall column; row percentage in the columns of vaccine refusal). * Age expressed as median and interquartile range; *p*-value obtained via the Mann–Whitney U test. ° or “daily caring of a frail individual”.

**Table 2:** Descriptive analyses of secondary outcomes, variables related to health and COVID-19, and relationship with vaccine refusal.

Characteristic	Overall Sample	COVID-19 Vaccine Refusal		
	(n = 1517) N (%)	No (n = 1327) N (%)	Yes (n = 186) N (%)	*p*-Value
Score of misclassification of fake news *	46.7 (40–56.7)	46.7 (40–53.3)	66.7 (50–70)	**<0.001**
Score of belief in conspiracy theories *	2.8 (2.2–3.4)	2.8 (2.2–3.4)	3.6 (2.8–4.4)	**<0.001**
**Suffering from a chronic disease**				
No	1240 (82)	1081 (87.2)	158 (12.8)	0.264
Yes	272 (18)	244 (89.7)	28 (10.3)	
**Having contracted SARS-CoV-2**				
No	1279 (84.6)	1124 (87.9)	154 (12.1)	0.575
Yes	232 (15.4)	201 (86.6)	31 (13.4)	
**Relative/close friend who had contracted SARS-CoV-2**				
No	392 (25.9)	327 (83.6)	64 (16.4)	**0.004**
Yes	1119 (74.1)	998 (89.2)	121 (10.8)	
**Inadequate Health Literacy (SILS)**				
No	1264 (83.5)	1116 (88.4)	147 (11.6)	0.073
Yes	249 (16.5)	209 (84.3)	39 (15.7)	
**Inadequate eHealth Literacy (eHEALS)**				
No	755 (58.4)	674 (89.3)	81 (10.7)	0.504
Yes	537 (41.6)	473 (88.1)	64 (11.9)	

*p*-value obtained via chi-squared test (significant *p*-values in bold). Figures expressed as frequency and percentage (column percentage in the overall column; row percentage in the columns of vaccine refusal). * Scores expressed as median and interquartile range; *p*-value obtained via the Mann–Whitney U test (significant *p*-values in bold). Abbreviations: eHEALS, eHealth literacy scale; SILS, single-item screener.

**Table 3:** Univariable and multivariable logistic regressions with vaccine refusal as outcome.

Outcome: Vaccine Refusal	Univariable Regressions		Multivariable Model	
	OR (95% CI)	*p*-Value	adjOR (95% CI)	*p*-Value
**Age**	0.99 (0.99; 1.01)	0.529	0.97 (0.96; 0.99)	**0.001**
**Female**	1.33 (0.90; 1.97)	0.153	1.30 (0.79; 2.15)	0.297
**Educational level: high school diploma or lower**	2.99 (2.07; 4.30)	**<0.001**	1.84 (1.16; 2.91)	**0.009**
**Background (study/work)**				
Health care	Ref.		Ref.	
Informatics	3.15 (1.67; 5.93)	**<0.001**	1.63 (0.73; 3.6)	0.232
Journalism	2.03 (0.44; 9.40)	0.366	0.63 (0.07; 5.78)	0.686
Other	2.91 (1.79; 4.72)	**<0.001**	1.50 (0.82; 2.74)	0.183
**Occupation**				
Worker	Ref.		Ref.	
Student	0.32 (0.16; 0.65)	**<0.001**	0.28 (0.12; 0.67)	**0.004**
Other	1.10 (0.75; 1.59)	0.631	0.73 (0.43; 1.24)	0.247
**Economic situation: insufficient/poor**	2.68 (1.94; 3.70)	**<0.001**	1.49 (0.97; 2.27)	0.067
**Relative/close friend working in health care**	0.68 (0.49–0.96)	**0.026**	0.74 (0.47; 1.14)	0.168
**Sharing the habitation with a frail individual °**	0.57 (0.37; 0.88)	**0.010**	0.5 (0.29; 0.86)	**0.012**
**Relative/close friend who had contracted SARS-CoV-2**	0.62 (0.45; 0.86)	**0.004**	0.77 (0.5; 1.18)	0.229
**Inadequate Health Literacy (SILS)**	1.42 (0.67; 2.08)	0.074	0.69 (0.4; 1.19)	0.182
**Score of belief in conspiracy theories**	2.41 (1.97; 2.94)	**<0.001**	1.82 (1.46; 2.27)	**<0.001**
**Score of fake news misclassification**	1.06 (1.05; 1.07)	**<0.001**	1.05 (1.03; 1.06)	**<0.001**

Significant *p*-values in bold. Abbreviations: adjOR, adjusted odds ratio; CI, confidence interval; OR, odds ratio; SILS, single-item screener. ° or “daily caring of a frail individual”.

**Table 4:** Univariable and multivariable logistic regressions with the score of fake news misclassification as outcome.

Outcome: Score of Fake News Misclassification	Univariable Regressions		Multivariable Model	
	**Coef. (95% CI)**	***p*-Value**	**adjCoef. (95% CI)**	***p*-Value**
**Age**	0.12 (0.08; 0.17)	**<0.001**	0.04 (−0.01; 0.09)	0.094
**Female**	0.08 (−1.53; 1.69)	0.922	−1.13 (−2.55; 0.3)	0.121
**Educational level: high school diploma or lower**	5.92 (4.60; 7.24)	**<0.001**	2.07 (0.79; 3.36)	**0.002**
**Background (study/work)**				
Health care	Ref.		Ref.	
Informatics	9.01 (6.53; 11.49)	**<0.001**	4.7 (2.33; 7.07)	**<0.001**
Journalism	11.04 (5.23; 16.85)	**<0.001**	10 (4.79; 15.22)	**<0.001**
Other	7.38 (5.85; 8.92)	**<0.001**	4.3 (2.78; 5.82)	**<0.001**
**Occupation**				
Worker	Ref.		Ref.	
Student	−1.55 (−3.59; 0.49)	0.136	2.11 (0.04; 4.19)	**0.046**
Other	5.93 (4.20; 7.66)	**<0.001**	3.7 (2.01; 5.4)	**<0.001**
**Economic situation: insufficient/poor**	5.85 (4.24; 7.47)	**<0.001**	2.7 (1.2; 4.2)	**<0.001**
**Relative/close friend working in health care**	−2.72 (−4.11; −1.33)	**<0.001**	−0.56 (−1.83; 0.7)	0.384
**Relative/close friend who had contracted SARS-CoV-2**	−3.17 (−4.71; −1.63)	**<0.001**	−1.92 (−3.3; −0.54)	**0.006**
**Inadequate Health Literacy (SILS)**	3.43 (1.58; 5.28)	**<0.001**	2.12 (0.44; 3.79)	**0.013**
**Inadequate eHealth Literacy (eHEALS)**	5.28 (3.94; 6.62)	**<0.001**	3.26 (2.00; 4.51)	**<0.001**
**Score of belief in conspiracy theories**	4.14 (3.43; 4.84)	**<0.001**	2.79 (2.11; 3.47)	**<0.001**

Significant *p*-values in bold. The model was also adjusted for vaccine refusal. Abbreviations: adjCoef, adjusted coefficient; CI, confidence interval; Coef, coefficient; eHEALS, eHealth literacy scale; SILS, single-item screener.

**Table 5:** Univariable and multivariable logistic regressions with the score of belief in conspiracy theories as outcome.

Outcome: Score of Belief in Conspiracy Theories	Univariable Regressions		Multivariable Model	
	**Coef. (95% CI)**	***p*-Value**	**adjCoef. (95% CI)**	***p*-Value**
**Age**	0.003 (0.001; 0.01)	**0.046**	0.002 (−0.002; 0.01)	0.426
**Female**	0.19 (0.07; 0.31)	**0.002**	0.15 (0.04; 0.27)	**0.008**
**Educational level: high school diploma or lower**	0.39 (0.29; 0.49)	**<0.001**	0.21 (0.11; 0.31)	**<0.001**
**Background (study/work)**				
Health care	Ref.		Ref.	
Informatics	0.27 (0.08:0.46)	**0.006**	0.04 (−0.15; 0.22)	0.704
Journalism	−0.03 (−0.47; 0.42)	0.912	−0.17 (−0.58; 0.25)	0.435
Other	0.21 (0.09; 0.33)	**0.001**	0.02 (−0.1; 0.14)	0.736
**Occupation**				
Worker	Ref.		Ref.	
Student	−0.16 (−0.31; −0.003)	**0.046**	−0.09 (−0.25; 0.08)	0.287
Other	0.06 (−0.07; 0.19)	0.353	−0.1 (−0.24; 0.04)	0.146
**Economic situation: insufficient/poor**	0.43 (0.31; 0.55)	**<0.001**	0.21 (0.09; 0.33)	**0.001**
**Having contracted SARS-CoV-2**	0.12 (−0.02; 0.26)	0.101	0.07 (−0.07; 0.20)	0.324
**Relative/close friend who had contracted SARS-CoV-2**	−0.09 (−0.21; 0.02)	0.109	−0.02 (−0.14; 0.09)	0.660
**Inadequate Health Literacy (SILS)**	0.19 (0.05; 0.33)	**0.006**	0.15 (0.02; 0.28)	**0.027**
**Inadequate eHealth Literacy (eHEALS)**	0.01 (−0.09; 0.12)	0.797	−0.15 (−0.25; −0.05)	**0.003**
**Score of fake news misclassification**	0.02 (0.02; 0.03)	**<0.001**	0.02 (0.01; 0.02)	**<0.001**

Significant *p*-values in bold. The model was also adjusted for vaccine refusal. Abbreviations: adjCoef, adjusted coefficient; CI, confidence interval; Coef, coefficient; eHEALS, eHealth literacy scale; SILS, single-item screener.

## Data Availability

All relevant data are within the paper. The database is available from the corresponding author on reasonable request.

## Supplementary Materials

The following supporting information can be downloaded at: https://www.mdpi.com/article/10.3390/ijerph19159350/s1. Supplementary Material S1 includes: Table S1: Descriptive analysis of the items of the score assessing misclassification of true and fake news; Table S2: Descriptive analysis of the items of the score assessing the level of agreement with conspiracy theories; Table S3: Characteristics of the sample and relationships with secondary outcomes.
