# Supplementary material for: Exploring the Relationship between COVID-19 Vaccine Refusal and Belief in Fake News and Conspiracy Theories: A Nationwide Cross-Sectional Study in Italy
Source: Int J Environ Res Public Health. 2022 Jul 30;19(15):9350. doi: 10.3390/ijerph19159350 (PMC9368531; doi:10.3390/ijerph19159350)
Supplement: Supplementary file 1 [file ijerph-19-09350-s001.zip › ijerph-1823287-supplementary.pdf]

Supplementary Material S1:

Table S1. Descriptive analysis of the items of the score assessing misclassification of true and fake news.

Item	Misclassification	
	Frequency	Percentage
Children are not contagious in case of a positive test for COVID-19	174	13.45
Vaccines do not provide sufficient protection against the COVID-19 disease because they have been developed too quickly and, therefore, they have not been studied enough	279	21.56
Being able to hold your breath for 10 seconds without coughing means that your respiratory system is not compromised and that you are not infected by SARS-CoV-2	328	25.35
Anti-SARS-CoV-2 vaccines provide the highest possible protection twenty-four hours after administration	316	24.42
Electromagnetic fields emitted by 5G antennas facilitate SARS-CoV-2 transmission	131	10.12
The increase in body temperature detected by thermal scanners makes it possible to detect with certainty whether or not a person is positive for SARS-CoV-2	84	6.49
Shoe soles definitely bring the virus into the house and can transmit the infection	84	6.49
Prolonged use of the mask can lead to carbon dioxide intoxication	381	29.44
Nasopharyngeal swab can cause brain damage	114	8.81
One should wait at least 72 hours before carrying out a molecular test (swab) after coming into close contact with an individual positive for SARS-CoV-2	314	24.27
The effectiveness of the mask is not affected by the length of the beard	889	68.70
Ibuprofen administration in patients who have developed COVID-19 significantly worsens the disease	711	54.95
Transmission of SARS-CoV-2 through contact with domestic animals is possible	135	10.43
Taking antibiotics prevents SARS-CoV-2 infection	170	13.14
Taking vitamins C and D prevents SARS-CoV-2 infection	899	69.47
Reducing meat consumption prevents SARS-CoV-2 infection	282	21.79
Taking a hot bath prevents SARS-CoV-2 infection	229	17.70
Ayurveda treatments prevent SARS-CoV-2 infection	402	31.07
Chili pepper consumption prevents SARS-CoV-2 infection	365	28.21
Garlic consumption prevents SARS-CoV-2 infection	341	26.35

Table S2. Descriptive analysis of the items of the score assessing the level of agreement with conspiracy theories.

Item	Median	Interquartile Range
Many very important things happen in the world, which the public is never informed about	3	3-5
Politicians usually do not tell us the true motives for their decisions	4	3-5
Government agencies closely monitor all citizens	2	2-3
Events which superficially seem to lack a connection are often the result of secret activities	2	1-3
There are secret organizations that greatly influence political decisions	2	1-3

Table S3. Characteristics of the sample and relationships with secondary outcomes.

Characteristic	Score of fake news misclassification		Score of belief in conspiracy theories	
	Median (IQR)	p-value	Median (IQR)	p-value
**Gender**				
Male	46.67 (40-56.67)	0.531	2.6 (2.2-3.2)	**0.004**
Female	46.67 (40-56.67)		2.8 (2.2-3.6)	
**Nationality**				
Italian	46.67 (40-56.67)	**0.002**	2.8 (2.2-3.4)	0.053
Other	53.33 (46.67-66.67)		3.2 (2.4-4)	
**Educational level**				
Diploma or below	50.00 (43.33-60.00)	**<0.001**	3.0 (2.4-3.8)	**<0.001**
University degree or above	45.00 (40.00-50.00)		2.6 (2.0-3.2)	
**Work or study background**				
Informatics	50 (43.33-63.33)	**<0.001**	3 (2.4-3.6)	**<0.001**
Health Care	40 (36.67-50)		2.6 (2-3.2)	
Journalism	46.67 (53.33-66.67)		2.6 (2-3.2)	
Other	50 (43.33-60)		2.8 (2.2-3.6)	
**Occupation**		**<0.001**		0.098
Worker	46.67 (40.00-56.67)		2.8 (2.2-3.4)	
Student	46.67 (40.00-53.33)		2.6 (2.2-3.2)	
Other	50 (43.33-66.67)		2.8 (2.2-3.6)	
**Economic situation**				
Excellent/adequate	46.67 (40-53.33)	**<0.001**	2.8 (2.2-3.4)	**<0.001**
Insufficient/poor	51.67 (43.33-63.33)		3 (2.6-4)	
**Family member in health care**				
Yes	46.67 (40-53.33)		2.8 (2.2-3.4)	
No	46.67 (43.33-56.67)	**<0.001**	2.8 (2.2-3.4)	0.217
**Chronic conditions**				
Yes	46.67 (43.33-56.67)		2.8 (2.2-3.6)	
No	46.67 (40-56.67)	0.672	2.8 (2.2-3.4)	0.694
**Living with a frail person**				
Yes	46.67 (40-56.67)		2.8 (2.2-3.6)	
No	46.67 (40-56.67)	0.255	2.8 (2.2-3.4)	0.759
**Positivity to SARS-CoV2**				
Yes	46.67 (40-56.67)		2.8 (2.2-3.6)	
No	46.67 (43.33-56.67	0.516	2.8 (2.2-3.4)	0.115
**Relatives or friends tested positive**				
Yes	46.67 (40-56.67)		3 (2.2-3.6)	
No	50 (43.33-60)	**0.001**	2.8 (2.2-3.4)	0.084
**SILS: adequate health literacy**	36.67 (40-56.67)	**<0.001**	2.8 (2-3.4)	**0.003**

**SILS: inadequate health literacy**	50 (43.33-60)		3 (2.4-3.6)	
**eHEALS: adequate ehealth literacy**	46.67 (40-60)	**<0.001**	3 (2.4-3.6)	0.526
**eHEALS: inadequate ehealth literacy**	50 (43.33-60)		2.8 (2.2-3.4)	

Figures expressed as median and interquartile range.
p-value obtained via the Mann Whitney U test (Kruskall Wallis test for work/study background and occupation).
Abbreviations: eHEALS eHealth Literacy Scale; SILS Single Item Screener
